# Histone Modification of Colorectal Cancer by Natural Products

**DOI:** 10.3390/ph16081095

**Published:** 2023-08-02

**Authors:** Zijun Geng, Meiqi Chen, Qixuan Yu, Shuoxi Guo, Tianli Chen, Da Liu

**Affiliations:** School of Pharmacy, Changchun University of Chinese Medicine, Changchun 130117, China; jimmyG_98@163.com (Z.G.); 15567958349@163.com (M.C.); yuqx0221@163.com (Q.Y.); shuoxiguo_1824@163.com (S.G.)

**Keywords:** epigenetics, colorectal cancer, histone modification, histone deacetylation and acetylation, histone phosphorylation, histone methylation and demethylation, natural products

## Abstract

Natural products play important roles in the pathogenesis of many human malignancies, including colorectal cancer, and can act as a gene regulator in many cancers. They regulate malignant cell growth through many cellular signal pathways, including Rac family small GTPase 1 (RAC1)/PI3K/AKT (α-serine/threonine-protein kinase), mitogen-activated protein kinase (MAPK), Wnt/β-catenin pathway, transforming growth factor-β (TGF-β), Janus kinase and signal transducer and activator of transcription (JAK-STAT), nuclear factor kappa-B (NF-κB), the Notch pathway, Hippo pathway, and Hedgehog pathway. In this review, we describe the epigenetic roles of several natural products, e.g., platycodin D (PD), ginsenoside Rd, tretinoin, Rutin, curcumin, clove extract, betulinic acid, resveratrol, and curcumin, in colorectal cancer, including their impact on colorectal cancer cell proliferation, apoptosis, invasion, migration, and anti-chemotherapeutic resistance. The aim is to illustrate the epigenetic mechanisms of action of natural products in cancer prevention and treatment, and to provide (1) a theoretical basis for the study of the role of epigenetics in influencing colorectal cancer; (2) new directions for studying the occurrence, development, and prognosis of colorectal cancer; and (3) new targets for treating and preventing colorectal cancer.

## 1. Introduction

Colorectal cancer (CRC) is one of the most common malignancies of the gastrointestinal tract, and its incidence is increasing annually [1]. According to cancer statistics from China and the United States for 2022, the age-standardized incidence and mortality rates of lung and CRC in the United States have decreased significantly, However, the incidence and burden of CRC in the global population are increasing. CRC is characterized by insidious onset, inconspicuous early clinical manifestations, difficult diagnosis, and extremely poor prognosis, and most patients with CRC are already in the middle to late stages or have metastatic symptoms at diagnosis. While surgical resection with radiotherapy is currently the main treatment method for CRC, it has no significant therapeutic effect.

CRC has a very complex development mechanism, and the traditional view is that the main mechanism of the tumor is uncontrolled cell growth and differentiation due to DNA sequence variation caused by oncogenic factors. As research has progressed in recent years, it has become apparent that abnormalities in regulatory mechanisms other than DNA sequence are more common in the tumor development process, leading to increasing research on epigenetics. Epigenetics differs from genetics in that heritable changes in gene function can occur, causing changes in phenotype but not in DNA sequence. Changes in epigenetic regulation are inherited along with the replicated DNA in the daughter cells. Epigenetic regulation plays a key role in the diagnosis and treatment of CRC. With the intensive study of human epigenetics, several epigenetic phenomena have been identified; the regulatory mechanisms include DNA methylation, histone modification, non-coding RNA regulation, chromatin remodeling, and nucleosome localization in five directions, among which histone modification has been studied the most frequently.

Epigenetic changes are crucial in CRC development and progression. The fact that epigenetic mechanisms are plastic (so-called epigenetic plasticity) offers an attractive option to restore specific changes in CRC [2]. Among them, epigenetic modulation by natural products plays a more promising role in CRC prevention and treatment; natural products are currently considered an important source of new antitumor drugs due to their low adverse effects, with the US Food and Drug Administration (FDA) progressively approving 175 small molecule anticancer drugs, 63% of which are natural chemical drugs or derived directly from natural products [3]. Natural products are the constituents or metabolites of animals, plants, insects, marine organisms, and microorganisms. They include many endogenous chemical components in human and animal cells, including proteins, peptides, amino acids, nucleic acids, various enzymes, monosaccharides, oligosaccharides, polysaccharides, glycoproteins, resins, colloids, lignans, vitamins, fats, oils, waxes, alkaloids, volatile oils, flavonoids, glycosides, terpenoids, phenylpropanoids, organic acids, phenols, quinones, lactones, steroids, tannins, antibiotics, and other naturally occurring chemical constituents.

A growing body of research suggests that epigenetic regulation by natural products is one of the pathways that play a key role in CRC pathogenesis. Many natural products play an important role in several life processes, such as inhibiting CRC cell proliferation, migration, and invasion; cell cycle arrest; and inducing CRC cell apoptosis. Several of the more obvious and less toxic natural products have the potential to be developed as anticancer or anticancer drugs. By acting on different epigenetic targets such as oncogenes (Ras and p53 genes) and histone deacetylases (HDACs) [4], these natural products can inhibit CRC cell growth, proliferation, migration, and invasion and increase their sensitivity to anticancer drugs to reduce drug resistance development, inhibiting CRC.

This review suggests that histone modifications caused by different natural products (e.g., resveratrol, E curcumin, and radicicol) can have a benign effect on CRC development and later chemotherapy, and may provide new directions for studying epigenetic regulation in CRC by natural products. In addition, natural products that mediate CRC development by mediating proteins and transcription factors downstream of the histone-modifying enzymes and the mechanisms of histone modifications are discussed.

## 2. The Role of Histone Modifications in CRC

CRC development is a complex process regulated by multiple genes and steps. It is often associated with uncontrolled cell proliferation and apoptosis, involving proto-oncogenes, oncogenes, mismatch repair genes, and modifier genes. It can be divided into the following categories based on its molecular mechanism: deficient DNA damage repair function, proto-oncogene and oncogene mutation, microsatellite instability, telomerase abnormal expression, and signal transduction disorder [5]. With research and progress in molecular biology, there are deeper studies on the mutation of proto-oncogenes and oncogenes. The key genes prone to mutation in CRC are the proto-oncogenes B-Raf proto-oncogene, serine/threonine kinase (BRAF), RAS family members (HRAS, KRAS, and NRAS), and phosphatidylinositol 3-kinase catalytic subunit type 3 (PIK3CA), the APC regulator of the WNT signaling pathway (APC), and tumor protein p53 (p53). When the original oncogene is mutationally activated, it can cause abnormalities in the signaling pathways it is involved in, causing malignant cell proliferation and metastasis and thereby promoting tumor development. Mutations in BRAF, RAS family members (including KRAS, NRAS, HRAS), or PIK3CA [6] may activate their downstream RAS-RAF-MAPK and PI3K-AKT-mTOR pathways. Mutations in these genes lead to sustained activation of downstream signal pathways, causing abnormal cell proliferation independent of epidermal growth factor (EGF) stimulation, ultimately leading to tumor development and continuous progression. The overexpression of proto-oncogenes promotes cancer cell growth. As an important oncogene, p53 can inhibit cellular damage and differentiation, and the expression of wildtype p53 protein inhibits abnormal cell proliferation by blocking the cellular G1/G0 phase and preventing the cell from entering the S-phase [7,8,9]. At the same time, the p53 protein can induce programmed cell death. It was found that by transfecting the p53 gene into tumor cell lines cultured in vitro, overexpression of the p53 protein caused tumor cell apoptosis [10,11].

Abnormal epigenetic alterations were first identified in CRC in 1982. Research has subsequently revealed an “epigenetic landscape” comprising a complex set of epigenetic regulatory mechanisms controlling gene expression in normal and cancerous tissues [12]. As research has progressed, it has become evident that there are multiple epigenetic patterns of aberrant expression in CRC, that different epigenetic modifications have various roles in CRC, and that epigenetic modifications are often involved in transcriptional regulation [13]. Epigenetic alterations are a series of heritable changes in gene expression without changes in DNA sequence. They can be divided into several overlapping mechanisms, including DNA methylation, histone modifications, microRNA dysregulation, chromosomal instability, and microsatellite instability.

Epigenetic alterations come in two forms. One involves epigenetic factors directly affecting epigenetic enzymes, changing their bioavailability in the cell. The other involves epigenetic factors interfering with biochemical pathways, changing the availability of the metabolites required to create the epigenetic label. Both forms may result in abnormal or inadequate alteration of epigenetic tag recruitment to non-specific promoters in a stochastic manner, ultimately producing epigenetic aberrations [14]. Among others, epigenetic factors indirectly affect the epigenome by first interfering with cellular signal pathways, including PI3K/AKT, MAPK, Wnt/β-catenin, TGF-β, JAK-STAT, NF-κB, Notch, and Hedgehog, which can lead to altered expression of growth factors, receptors, and ion channels, resulting in non-homeostatic cellular processes. This process may in turn lead to alterations in the state of the transcriptional machinery and its bioavailability in the cell, which has implications for CRC development and progression. Among others, the Wnt/β-catenin signal pathway is considered the initial CRC marker (i.e., high expression of Wnt-related genes). The Wnt signaling pathway includes the adenomatous polyposis coli (APC) protein and the low-density lipoprotein receptor-related protein 5 (LRP5). Among them, β-catenin is a key component of the Wnt signal pathway, which plays an important role in maintaining intercellular adhesion and the morphological structure of adjacent tissues [15]. Histone modifications can mediate the transcription and translation of proteins associated with the Wnt signaling pathway by acetylating N-methylated histone tails, thereby inhibiting CRC cellular replication.

As a form of epigenetic regulation, histone modifications have been studied in treating and preventing CRC, mainly by modifying chromatin structure to regulate gene expression in CRC. Histones are highly conserved proteins comprising five core protein types (H1, H3, H2A, H2B, and H4) [16] that are usually covalently modified at their N-terminal tail, exposing the surface of the nucleosome to exert a regulatory effect on gene expression. Because different histones (H3 and H4) can undergo different types of modifications at different amino acids (7Lys and 2Ser at the end of H3; 5Lys and 1Ser at the end of H4), including acetylation, methylation, phosphorylation, ubiquitination, glycosylation, poly-ADP-ribosylation, carbonylation, and biotin(acyl)ation of lysine residues, they are all basic elements of histone modifications [17]. Histone modifications are catalyzed by enzymes such as histone methyltransferases (HMTs), demethylases (HDMs), acetyltransferases (HATs), and HDACs. They can act on related target proteins and genes downstream of histone-modifying enzymes to influence the activation and silencing of signal pathways, thereby exerting epistatic regulation.

## 3. Histone Methylation Regulates CRC

**Figure 1:**
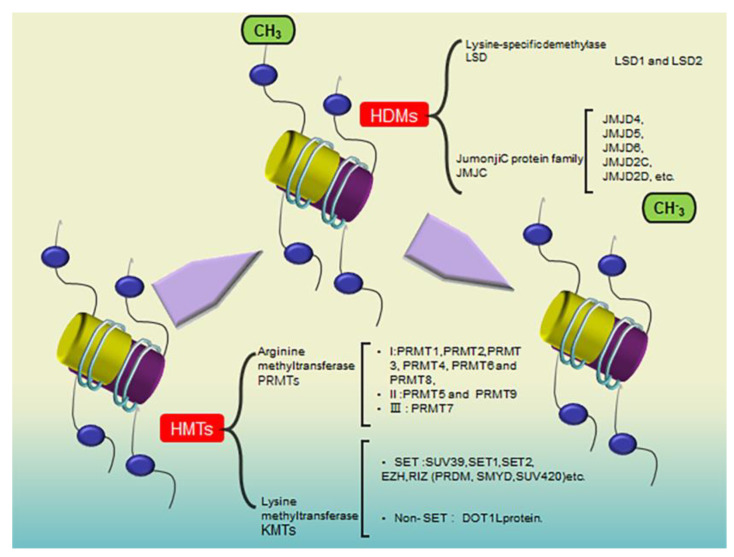
Histone methylation mechanisms: the way histone methylation transferase (HMTs) and histone demethylation (HDMs) enzymes act on histones and the classification of HMTs (arginine methyltransferase (PRMTs) and lysine methyltransferase (KMTs)) and HDMs (lysine−specific demethylase (LSD) and JumonjiC protein family (JMJC)).

Histone methylation is a post-translational modification of protein side chain amino acids to acquire different numbers of methyl groups as methyl donors using S-adenosylmethionine catalyzed by various methylation enzymes [16]. It is considered a marker of chromatin activity. The main methylation modification sites are the lysine and arginine residues of histones H3 and H4, as shown in Figure 1. A single lysine or arginine can be modified with up to three methyl groups. Depending on the amino acid residues modified, HMTs can be divided into arginine and lysine methyltransferases. Histone arginine methylation is a common post-translational modification, and abnormal histone arginine methylation is closely associated with carcinogenesis and metastasis [18]. HMTs and HDMs mainly catalyze histone methylation. There are >50 methylesterases with HMT activity and >24 HDMs.

Histone and DNA methylation can act together to induce tumors by silencing oncogenes. A frequently mutated gene in CRC is the tumor suppressor gene p53, known as the guardian of the genome [19]. Various stress signals can activate p53, such as DNA damage or oncogene activation. When activated, it mediates various cellular responses, including DNA repair, cell cycle arrest, senescence, apoptosis, ferroptosis, stem cell reprogramming, invasive metastasis, autophagy, metabolism, and cell death [20,21]. P53 mainly acts as a transcription factor, controlling the expression of hundreds of target genes [22]. Methylation modification of histones tightens chromatin and inhibits gene expression, while HDMs antagonize this process. The histone lysine methyltransferases SET domain containing 9, histone lysine methyltransferase (Set9), SET and MYND domain containing 2 (SMYD2), and SET domain containing 8/histone lysine methyltransferase (Set8) have been found to methylate the p53 protein and regulate its function. Its methylation sites are K372, K370, and K382, respectively [23,24,25]. Histone lysine-specific demethylase 1 (LSD1) was found to inhibit p53 function by specifically demethylating its K370 site, leaving CRC cells uninhibited and able to continue proliferating and differentiating [26]. 

Histone methylation was considered irreversible until LSD1 was discovered in 2004, which confirmed that histone methylation was a reversible genetic marker [27]. Studies have shown that LSD1 has an important role in the progression of human cancers, and research on LSD1 inhibitors has emerged as a new target for therapy [28,29]. HMTs can catalyze the methylation of the promoter region of the E-cadherin (ECAD) gene at histone H3 lysine 27 (H3K27) or lysine 9 (H3K9) [30], promoting the epithelial–mesenchymal transition (EMT) process in tumor cells.

Arginine methylation transferases (PRMTs) can affect the expression of cancer-related genes by methylating histone arginine residues. Curcumin can reduce the mRNA and protein levels of PRMT5 to inhibit cancer cell proliferation. HMT enhancer of zeste-2-polycomb repressive complex 2 subunit (EZH2) was found to promote tumor proliferation and transformation when overexpressed in various tumors. The polycomb repressive complex (PRC) repressed the expression of oncogenes by regulating the distribution of H3K27me3 across the genome. Cadherin transcription promotes the EMT process in CRC cells [31]. 

HDM lysine demethylase 4D (KDM4D), which belongs to the JMJD2 subfamily of HDMs containing the JMJC structural domain, can be recruited to the β-catenin promoter to induce β-catenin transcription and can act as a coactivator to enhance the expression of β-catenin target genes. KDM4D interacts with LSD1 to synergistically remove monomethyl dimethyl and trimethyl groups from H3K9 [32]. H3 lysine 4 (H3K4) methylation is usually associated with gene activation and transcriptional prolongation. It is mediated by HMTs such as SET domain containing 1A, histone lysine methyltransferase (SET1), and SET domain containing 7/histone lysine methyltransferase (SET7/9). In contrast, H3K9 is usually associated with gene repression, and is catalyzed by HMTs such as SUV39H1, G9a, and SETDB1/ESET [33]. As an HMT that promotes cancer development by repressing oncogenes, Drosophila zeste homolog 2 (EZH2) is a key marker of CRC. It is responsible for the methylation of histone H3K27 [34]. Among the many EZH2 inhibitors, CPI-169 and GSK126 have similar oncogenic effects [35]. Treating mice loaded with KARPAS-42 xenografts with both CPI-169 and GSK126 effectively reduced the level of H3K27 trimethylation, causing tumor regression. Another inhibitor [36], EPZ011989, showed significant tumor suppression in a mouse model with human B-cell lymphoma. PF-06726304 inhibited both wildtype EZH2 and Y641N-mutated EZH2 [37].

Natural products provide various chemical scaffolds with unique activity and relatively mild toxicity. Natural products with LSD1 inhibitory activity have been identified, including baicalin, resveratrol, and guanylic acid (GGA). Baicalin was the first LSD1 inhibitor discovered to have a non-covalent bond to the flavonoid group. Resveratrol, an irreversible LSD1 inhibitor, is believed to inhibit LSD1 activity in cells in vitro by directly binding to LSD1 [28]. GGA and its derivatives inhibit LSD1 activity by disrupting the protein–protein interaction between LSD1 and H3K4me2, leading to transcriptional neurotrophic receptor tyrosine kinase 2 (NTRK2) upregulation in SH-SY5Y cells [38]. The oligopeptide transporter solute carrier family 15 member 1 (SLC15A1) is an intestine-specific transporter protein, and DNA methyltransferase 1 (DNMT1) mediates hypermethylation of the proximal promoter region of the PEPT1 gene, which in turn leads to transcriptional repression and increased sensitivity of CRC to anticancer drugs [39]. 

Other natural products may complete histone methylation by regulating epistasis factors. For example, kaempferol reduces A4CT2 methylation levels by inhibiting the expression of DNA methyltransferases (DNMTs) in HCT-116 and HT-29 colon cancer cells, thereby promoting gene transcription. Kaempferol significantly downregulated the expression of key genes in the Wnt signal pathway downstream of A4CT2 [40] and the β-catenin signaling pathway in HCT-116 cells, thereby blocking the cells in the G1 phase and inducing apoptosis [41]. Rhubarbic acid can exert anti-CRC effects by inhibiting cell proliferation, promoting apoptosis, and inhibiting invasion and metastasis through the STAT3 signaling pathway [42]. PD can upregulate the activity of caspase 3 (CASP3) and shear its substrate PARP, inducing SW620 cell death [43]. Radicicol could inhibit β-catenin entry into the nucleus to disrupt the Wnt/β-catenin signaling pathway, reduce downstream target gene LEF/TCF expression, inhibit cell transcription and translation, cause G1 phase block in colon cancer HT29 and SW480 cells, and inhibit cell proliferation in a dose- and time-dependent manner [44]. Geniposide, shikonin, costunolide, asterolide, ursolic acid, and curcumin inhibit CRC development or induce its apoptosis (Table 1).

## 4. Histone Acetylation Regulates CRC

**Figure 2:**
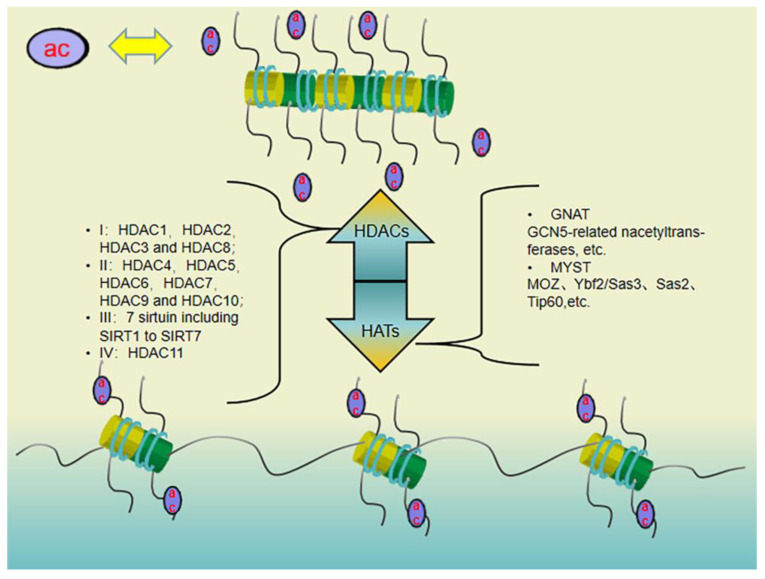
Histone acetylation mechanism: the mode of action of HATs and HDACs and the classification of HATs (e.g., GNAT, MYST, MOZ, Ybf2/Sas3, Sas2, and Tip60) and HDACs (I, II, III, and IV), “ac” means Acetyl in Figure 2.

Histone acetylation refers to the addition of acetyl groups carried by acetyl coenzyme A to histone residues in the presence of HATs. Histone acetylation usually occurs on lysine residues at positions 9, 14, 18, 23, 27, 36, and 56 of H3 and positions 5, 8, 12, and 16 of H4. Histone acetylation counteracts the positive charge carried by these residues, increasing repulsion from DNA and leading to a looser chromatin structure, thereby activating gene expression. The histone acetylation level is regulated by the dynamic balance of HAT and HDAC enzymatic activities [59], as shown in Figure 2. Histones can be deacetylated by HDACs, allowing them to carry a positive charge again, which enhances the interaction between histones and negatively charged DNA, leading to a tighter chromatin structure and consequent transcription repression. Eighteen HDACs have been identified in humans; based on their homology to yeast HDACs, they can be classified into four classes: class I (HDAC1, HDAC2, HDAC3, and HDAC8), class II (HDAC4, HDAC5, HDAC6, HDAC7, HDAC9, and HDAC10), class III (sirtuin, including SIRT1-SIRT7), and class IV (HDAC11).

Sirtuins 1 to 7 (SIRT1-SIRT7) are HDACs essential for normal cell proliferation and growth. CRC development is closely associated with HDACs, with HDAC1 accounting for 36.4%, HDAC2 for 57.9%, and HDAC3 for 72.9% of CRC cases. HDACs are a family of proteins responsible for removing acetyl groups from the N-acetyl group of histone lysine residues [60,61]. Abnormal expression of these genes leads to cell cycle arrest, chemosensitization, and apoptosis. In addition, during cellular stress HDAC1 can deacetylate the tumor suppressor p53 [62], reducing the cellular stress response [63,64].

Histone H3 lysine 27 acetylation (H3K27ac) [65] is generally enriched in genes regulating cell proliferation and differentiation [66]. H3K27ac is a current marker of transcriptional activation of gene expression [67,68]. In CRC cells, H3K27ac activates the expression of lncRNAEIF3J-AS1 and promotes cell proliferation. HDAC2 expression in CRC metastatic tissues is low, and is closely associated with the survival of patients with CRC. Low HDAC2 expression promotes EMT and CRC metastasis. By binding to the transcription factor SP1, HDAC2 is “anchored” to the H19 promoter region and catalyzes the deacetylation of histone H3K27 to suppress H19 expression. HDAC2 has a negative regulatory effect on CRC metastasis. Low HDAC2 expression promotes EMT and rectal cancer metastasis by upregulating H19/MMP14 [69]. 

Chen [70] found that the novel HMT SETDB1 was highly expressed in most CRC samples and cell lines. A review of studies on the relationship between histone modifications and cancer recently found that the acetylase activity of hMOF, an important acetyltransferase of human histone H4 lysine 16 (H4K16), increased its acetylation. Mitani et al. [71] studied human colon and rectal cancer cell lines, finding that histone acetylation was significantly upregulated in these cancer cells compared to normal cells. The dynamic balance of histone acetylation in the distal promoter region of the PEPT1 gene is maintained by HDAC1, which is responsible for the deacetylation of histone H3 lysine 18 acetylation (H3K18Ac) and H3K27Ac, and P300, which is responsible for catalyzing H3K18Ac and H3K27Ac to enhance the sensitivity of CRC to anticancer drugs [72,73]. 

HDAC2 knockdown promotes the expression of CRC EMT marker genes. HDAC2 is negatively regulated for CRC metastasis. Low HDAC2 expression promotes EMT and rectal cancer metastasis by upregulating H19/MMP14 [74,75]. HDAC7 is a member of the HDAC class IIa family, which is an epigenetic regulatory AKT [76], also known as protein kinase B (PKB), a serine/threonine protein kinase with a molecular weight of approximately 60 kDa [77]. AKT regulates several biological processes, including cell survival, proliferation, growth, and glycogen metabolism. The AKT signal pathway is closely associated with the development of many diseases, including malignancies. AKT signal pathway activation has been reported to be inhibited by targeting HDAC7 to suppress tumor development. P21 (CDKN1A) is an important member of the cyclin-dependent kinase inhibitor family that coordinates the cell cycle by inhibiting the activity of the cyclin-dependent kinase (CDK) complexes. Inhibition of AKT signal activation leads to increased p21 expression, leading to cell cycle inhibition. B-cell leukemia/lymphoma 2 (BCL2) [78], an oncogene with a significant inhibitory effect on apoptosis, is a downstream target gene of the AKT signal pathway. Inhibition of AKT signal pathway activation and reduced BCL2 expression promotes apoptosis.

HDAC7 knockdown has been found to increase ATF3 expression, which decreases AKT signaling pathway activation, increases p21 expression, and inhibits cell proliferation. In the colon cancer cell line LS174T, the HDAC inhibitors (HDACis) TMP269 and SAHA decreased HDAC7 levels, leading to upregulation of p21 [79], ATF3, and caspase 8 (CASP8) expression and downregulation of BCL2 expression. Downregulated BCL2 expression inhibited LS174T cell proliferation and promoted apoptosis [80].

HDACis inhibit tumor cell proliferation and survival. Their inhibition of HDAC activity enables HATs to increase acetylation levels, turning regions initially unable to engage with promoters into potential or novel target sites for transcription factors, allowing normal expression of oncogenes and thereby inhibiting tumor cell growth and proliferation and promoting apoptosis or necrosis [81]. The HDACi structure usually comprises three parts: a cap structure that interacts with the edge of the HDAC active pocket, a zinc ion binding group (ZBG), and a linker arm that is responsible for the linkage between the cap structure and the ZBG and can interact with the hydrophobic channel in the active site. The primary traditional ZBGs are iso-hydroxamic acid, benzamide, carboxylic acid, and mercaptan. HDACis containing these ZBG structures have been approved for marketing by the FDA or are in clinical trials. Iso-hydroxamic acid is the most widely used ZBG.

Trichostatin A (TSA, the first HDACi on the market), suberoylanilide hydroxamic acid (SAHA) [82], belinostat (PDX101), panobinostat (LBH589) [83], and quisinostat (JNJ-26481585) along with certain carboxylated HDACis such as sodium valproate [84], sodium butyrate, and sodium phenylbutyrate [85] and sulfhydryl groups such as romidepsin can significantly inhibit HDAC1, 2, and 4, and have weaker inhibitory activity against HDAC6 [86]. Several candidate sulfhydryl HDACis have been reported but have not yet entered clinical studies [87]. The core structure of thujaplicins, cycloheptatrienone (tropolone), has been designed and synthesized as a series of HDACis [88,89]. Butyrate is a class I and II HDACi that mainly disrupts the HDAC enzymatic activity region. TSA is a class of hydroxamic acid HDACi that mainly inhibits class I and II HDACs [90], with potent anticancer effects when combined with cisplatin [91,92]. 

The FDA has approved a number of hydroxamic acid inhibitors for cancer treatment, including the well-known SAHA and panobinostat [93]. Romidepsin (FK-228) is the main cyclic peptide inhibitor, directly inhibiting HDAC1 and HDAC2, which has been reported to inhibit PI3K activity as well [94]. The first two have been shown to have tumor-inhibiting effects in vitro, while mangosteen has shown antitumor properties in vitro [95]. Curcumin is already in clinical trials for cancer treatment [96]. Other small molecule inhibitors, such as C646 (a specific inhibitor of p300), have been shown to inhibit the proliferation of various cancer cells in vitro [97].

Several natural products have HDACi effects, such as radicicol [98], which can exert antitumor effects by inhibiting HDAC activity, and safranin, which can increase histone H3 and H4 acetylation. Treating A549 cells with safranin decreased HDAC mRNA and protein levels, leading to cell growth arrest and morphological changes. The inhibitory effect of flavopiridol on HDACs was associated with the downregulation of oncogenes (e.g., tumor necrosis factor [TNF], matrix metalloproteinase 2 [MMP2], and matrix metalloproteinase 9 [MMP9]), upregulation of oncogenes (e.g., p21 and p53), and positive regulation of the Bcl2/Bax family proteins in A549 cells. It triggered the cysteine cascade apoptotic pathway and promoted apoptosis in A549 cells. Resveratrol significantly inhibited class I and II HDACs and enhanced HAT activity [99]. Other natural products may be indirectly involved in histone acetylation modifications by modulating different epigenetic regulators. For example, the Salvia officinalis extract dihydrotanshinone could inhibit colon cancer cell proliferation by downregulating the expression of β-catenin and/or c-Myc, both of which are proteins downstream of the HDAC1/HIF1α/VEGFA signaling pathway, to inhibit Wnt/β-catenin signaling [100].

## 5. Histone Phosphorylation Regulates CRC

**Figure 3:**
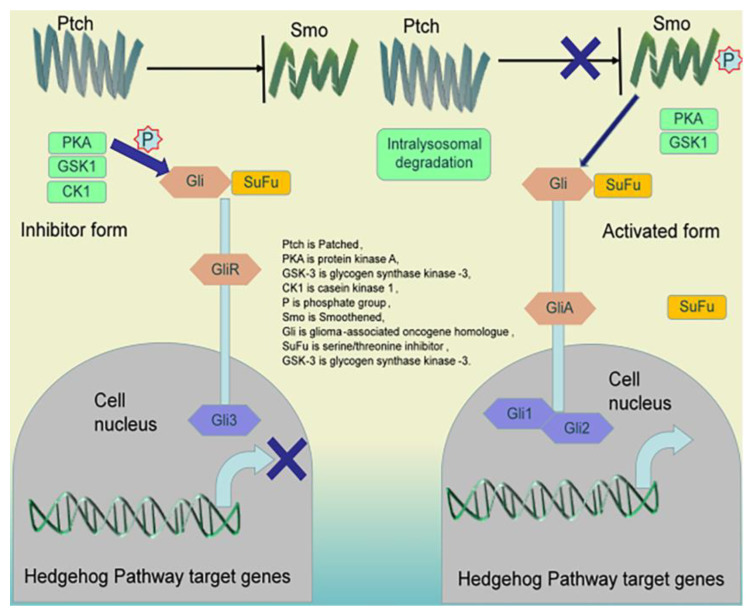
Histone phosphorylation regulates CRC. Phosphorylation of signal proteins usually causes the sequential phosphorylation of downstream proteins, creating a phosphorylation cascade. Protein phosphorylation mainly focuses on tyrosine, serine, and threonine residues in the peptide chain that have free hydroxyl groups and are uncharged. When phosphorylated, the proteins have an electrical charge, leading to structural changes and further changes in protein activity.

Histone phosphorylation modifications occur mainly on two amino acids, namely, serine (including threonine) and tyrosine. Serine phosphorylation activates the protein’s activity, primarily its enzyme activity. In contrast, tyrosine phosphorylation, in addition to its role in deactivating and activating the protein’s activity, has a more important function facilitating its interactions with other proteins to form a multiprotein complex, which further facilitates its phosphorylation [101], as shown in Figure 3. Therefore, tyrosine phosphorylation and the formation of multiprotein complexes represent the basic cell signaling mechanism; almost all peptide cell growth factors activate cells and stimulate cell growth through this pathway. Therefore, the enzymes that catalyze protein tyrosine phosphorylation, called tyrosine kinases, are key proteins in the signal transduction machinery and control of cell growth [102]. Tyrosine kinases and protein tyrosine phosphorylation play a decisive role in tumourigenesis and growth as well. Many antitumor drugs have been developed to target such molecules. 

Phosphorylation modifications often occur at threonine and serine residues of histones. Histone phosphorylation is associated with DNA damage and repair, and can increase the negative charge of the target proteins [103,104]. Histone phosphorylation leads to charge rejection between histones and DNA, potentially loosening DNA binding to histones, leading to decreased nucleosome stability, increased exposure of chromatin DNA, and enhanced chromatin accessibility. Phosphorylation is mainly involved in the activation of cellular signal pathways. Activation of the ERK-MAPK and p38-MAPK pathways induces Ser-10 phosphorylation of histone H3, which plays an important role in activating eukaryotic gene transcription [105]. Activation of the ATM/ATR and DNA-PK pathways induces Ser-10 phosphorylation of histone H2A variant H2AX, which is involved in DNA damage repair. In signaling, one group is phosphorylated by protein kinases and covalently binds phosphate groups provided by ATP, while the other binds GTP in response to the signal, usually replacing GDP with GTP. Both intracellular signaling proteins share the ability to be activated by acquiring one or more phosphate groups when the signal is received and inactivated by their removal when the signal is diminished. 

In the signal relay network, phosphorylation of one signal protein usually causes downstream proteins to be phosphorylated in turn, forming a phosphorylation cascade [106]. Protein phosphorylation is mainly concentrated on tyrosine, serine, and threonine residues in the peptide chain, which have free hydroxyl groups and are uncharged, which is the mechanism of protein phosphorylation [107]. Cinobufagin was intraperitoneally injected into mice with in situ human colon cancer SW480 cell tumors daily. After three weeks, mTOR phosphorylation was inhibited and HIF1α expression was downregulated in the tumor, blocking the endothelial mTOR/HIF-1α pathway and creating a microenvironment in which the tumor vasculature is not easily generated, triggering the apoptosis of vascular endothelial cells and inhibiting neoplastic tumor and vessel formation [47].

Acetylharpagide is expected to be a new candidate for treating tumors. β-catenin phosphorylation by the destruction complex is reduced, resulting in a large accumulation of β-catenin protein in the cytoplasm. The excess β-catenin is transferred from the cytoplasm to the nucleus, where it binds to intranuclear transcription factors such as T-cell transcription factor/lymphoid enhancement factor (TTF/LEF) and other intranuclear transcription factors to activate downstream target genes such as c-Myc and cyclin Dl (CCND1) [108]. Betulinic acid inhibits colon cancer cell growth and metastasis by regulating the TGF-β/Smad signaling pathway. It causes Smad protein receptor phosphorylation [109]. ANP32A is highly expressed in CRC tissues, correlating with their low differentiation, and is accompanied by the over-activation of AKT and reduced phosphorylation of p38. Molecular biology experiments have confirmed that knocking down ANP32A inhibits CRC cell proliferation, inducing over-phosphorylation of p38 and dephosphorylation of AKT [110].

Fan Ruolan and Chen Hailan found that C. rhamnose could bind to rhSTAT3 protein and inhibit STAT3 phosphorylation at the Tyr705 site, with significant anti-CRC activity [111]. Curcumin, a natural compound derived from natural products of the ginger and tannin families, inhibited the survival of human colon cancer HCT116 cells. Curcumin may slow down or even inhibit the development of distant metastasis by inhibiting the EMT of HCT116 cells induced by TNF-α through downregulation of the NF-κBp65 protein [112]. Curcumin inhibited NF-κB’s cytokine-induced DNA binding activity, RelA nuclear translocation, IκBα degradation, IκB Ser32 phosphorylation, and IκB kinase activity, thereby blocking NF-κB-induced kinase and IKK upstream signaling and inhibiting NF-κB activity [113]. 

The drug-containing serum of the Jianshuang Cancer Control Formula inhibited human colon cancer HCT116 cell proliferation [114], which was associated with the regulation of the ANP32A/p38/AKT signaling pathway by inhibiting ANP32A expression, increasing p38 phosphorylation, and inhibiting AKT phosphorylation [115]. Platycodon saponin, one of the active antitumor components in the Chinese medicine Platycodon, inhibits colon cancer cell growth by downregulating the expression of CCND1, c-Myc, and CDK6, blocking cells in the G1 phase, by inducing apoptosis by shear activation of pro-CASP3 and PARP, and by inhibiting JNK, p38 activation, and ERK1/2 dephosphorylation [116]. Acetylharpagide, a cyclic enol ether terpene compound in the natural plant genus Tendrils, inhibits HCT116 cell growth mainly through the Wnt/β-catenin signal pathway.

**Figure 4:**
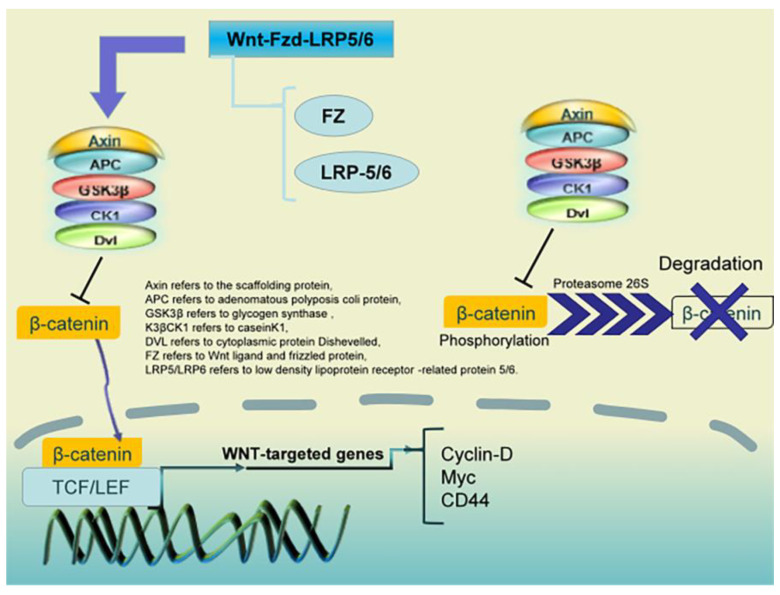
Mechanism of action of the Wnt signaling pathway: The major Wnt signal pathway components include the Wnt family of secreted proteins, the Frizzled family of transmembrane receptors, CK1, Deshevelled, GSK3, APC, Axin, β-catenin, and the TCF/LEF family of transcription factors.

β-catenin has been identified as a promising target for cancer prevention and treatment. Many natural products can act as β-catenin inhibitors, mainly by regulating phosphorylation, promoting ubiquitination and proteasomal degradation, and inhibiting nuclear translocation. Natural product inhibitors have shown good preventive and therapeutic effects in various in vitro and in vivo tumor models [117]. According to the research statistics in recent years, most (>90%) CRCs show activation of the classical Wnt pathway, as shown in Figure 4. The aberrant activation of Wnt/β-catenin, as one of the classical Wnt signal pathways, plays a crucial role in CRC development. The aberrant activation of this pathway is usually accompanied by the stabilization of β-catenin and a large accumulation of β-catenin in the nucleus [118]. This observation has been clinically demonstrated by the accumulation of β-catenin in the nucleus in >80% of patients with CRC [119]. In addition, clinical results have shown that the poor prognosis of patients with CRC is closely associated with high levels of cytosolic β-catenin [26,120]. Natural products act on Wnt signal pathway proteins to ameliorate CRC proliferation (Table 2).

## 6. Discussion

CRC is a malignant disease with the highest mortality rate of oncological diseases worldwide. It is treated only by surgical resection with radiotherapy, which remains ineffective. In the last decade or so, the clinical status of natural products as preventive and therapeutic agents has become a focus of attention due to their easy availability, low cost, high body tolerance, low toxicity, and diverse biological functions. Indeed, up to 70% of clinical drugs are derived from them. In recent years, epigenetic therapies have been increasingly accepted as effective treatments for cancer and various other diseases. Therefore, the epigenetic mechanisms of natural products have been used to investigate their preventive and therapeutic roles in CRC, where natural products provide a holistic approach to treating CRC.

With a growing understanding of the fundamental role of epigenetics in CRC initiation, progression, recurrence, and chemoresistance, natural products are very strong candidates for developing new drugs for cancer treatment. However, there remain limitations to using these natural products as chemotherapeutic agents, particularly regarding the range of effective concentrations of single natural product chemicals in preliminary in vitro models, making it difficult to define the most effective and biocompatible concentrations in animal models. In addition, in most cases the in vivo results are not as promising and encouraging as those observed with in vitro studies; therefore, they do not qualify for clinical trials. In addition, due to natural products’ confounding nature and reactivity, a thorough analysis of their appropriate validation and administration routes is required. Moreover, the mechanism of action of specific epigenetic factors needs further experimental validation. However, natural products continue to occupy a surprising amount of space in epigenetic drug development.

Understanding the mechanisms and collating the interactions between the various natural product chemicals to understand how they target the multi-step carcinogenesis process can help in developing personalized epigenetic regimens for patients. Drugs that target epigenetic alterations can overcome the immunogenicity of the tumor microenvironment, making these tumors sensitive to chemotherapy. Manipulating the epigenomic landscape through natural products would be an effective way to target immune-related pathways. Establishing this transcription factor pathway of histone modifications, such as acetylation, for key genes using natural products would be fascinating. To date, natural products have shown great potential in sensitizing CRC cells to conventional therapies, though this may lead to complex and unwanted off-target effects. There are many possible reasons for this issue, with a prominent drawback being that targetable enzymes/proteins with multiple biochemical functions in the normal cellular state may be disrupted upon treatment with these natural products. Indeed, detailed knowledge of the role of each protein complex/enzyme in the normal epigenetic landscape is essential for further consideration of natural products in epigenetic therapies. Therefore, natural product epigenetic modulators need to be considered more broadly in order to maximize their clinical benefit for patients with CRC, which may help expand their therapeutic potential in treating and preventing CRC.

## 7. Materials and Methods

Data sources and search strategy: in the PubMed database with “Colorectal cancer”, “Natural Products” AND “Epigenetics” OR “Histone modification” OR “Histone deacetylation and acetylation” OR “Histone phosphorylation” OR “Histone methylation and demethylation” as the subject of the search, for which there was no time limit and the search remained open until 31 May 2023. 

Literature Screening Criteria: ① Inclusion Criteria: articles investigating natural products to epigenetically regulate the treatment and prevention of colorectal cancer; ② Exclusion Criteria: non-natural products, non-epigenetic regulation, old or incomplete information, sketchy description of mechanism of action, unclear description of test method, sketchy description of test index, inconsistency between the description of test index and results, conference papers, the same experiment or the same batch of experiments. 

Data extraction and processing: End Note x20 standard edition was used for literature management. The preliminary search returned 287 PubMed and 211 Web of Science documents, for a total of 498 documents. After checking, 189 were excluded, leaving 309 documents. After reading through the titles and abstracts, 103 documents with incompatible topics were deleted, leaving 206 documents. After reading the full text and deleting 68 articles that did not meet the inclusion criteria, 138 articles were finally included, among which many had only abbreviations for gene and protein descriptions with reference to “the resource for approved human gene nomenclature” for standardizing the names, types, etc. of genes and proteins. Excel 2019 software was applied to enter the information of article year, author, title, impact factor, signaling pathway, epigenetic regulation mode of action, colorectal cancer treatment detection indexes, etc., to establish the data file of natural product epigenetic regulation of colorectal cancer, and the entered data were sorted, statistically and analytically analyzed, and quantitatively summarized.

## Figures and Tables

**Table 1:** Natural products and their pathways of action for treating CRC.

Serials	Natural Products	Cas	Origin	Signal Pathways	References
1	Butane	4478-93-7	Thioglucoside is hydrolyzed by black mustard enzyme in plants	Inhibition of HDACs activity	[42]
2	Platycodin D	58479-68-8	Extract of Platycodon grandiflorus, family Platycodonaceae	p38	[43]
3	Triptolide;	38748-32-2	The roots, leaves and flowers of the Weeping Vine	PI3K/Akt	[44]
4	pristimerin;	1258-84-0	One of the active ingredients of the roots of the Weeping Maple	Wnt/β-catenin	[45]
5	Peiminine;	18059-10-4	*Phyllostachys* spp. extract	Hedgehog	[46]
6	Rutin	153-18-4	Rutinoside, a natural flavonoid glycoside	Notch	[47]
7	Hederagenin;	465-99-6	Herb extracts such as Willingham	EMT	[48]
8	Andrographolide	5508-58-7	The active ingredients of Andrographis paniculata, family Siracaceae	Hedgehog	[47]
9	Sinomenine;	115-53-7	The roots and stems of the green vine of the family Fabaceae	NF-κB	[49]
10	Tenacissoside G;	191729-43-8	One of the components of the total saponin of Tongguan vine, family Lauraceae	ATM-CHK2-p53	[50]
11	Desacetylcinobufagin;	4026-95-3	One of the main bioactive components of the skin and parotid gland behind the ear of the Chinese toad	Wnt/β-catenin	[51]
12	Magnoflorine	6681-18-1	Apomorphine-like alkaloids in medicinal plants of the family Fabaceae	ROS/KRAS/AMPK	[52]
13	Geniposide;GEN	24512-63-8	A cyclic alkane glycoside extracted from Gardenia jasminoides, family Cyperaceae	Akt/MDM2/p53	[53]
14	shikonin, Shi	517-89-5	Comfrey root extract	Oxidative stress	[54]
15	Costunolide, CT	553-21-9	Sesquiterpene lactone compounds in the Chinese herb Mucuna pruriens	Up-regulation of Caspase-3 and Caspase-9 protein expression	[55]
16	Asterolide	73069-14-4	Extracts of natural products such as Atractylodes macrocephala	Inhibition of tumour cell stemness	[52]
17	Ursolic acid	77-52-1	Extracts of the leaves of Mignonette and Bearberry of the family Rhododendronaceae	Hedgehog, Wnt/β-catenin	[56]
18	Curcumin	458-37-7	A polyphenolic compound in the rhizome of Turmeric, family Gingeraceae	NF-KB, EMT, Wnt/β-catenin	[57]
19	5,6-Dihydro-9,10-dimethoxybenz	141433-60-5	An isoquinoline alkaloid from the buttercup plant Huanglian	EGFR, NF-κB, Wnt/β-catenin	[58]

**Table 2:** Natural products acting on the Wnt/β-catenin signal pathway.

Serials	Cas	Natural Products	Categorisation	Origin	Related Targets	References
1	520-18-3	Kaempferol	flavonoid	Polyphenol anti-inflammatory antioxidants in fruits and vegetables	Inhibited the expression of DNMTs and HDACs in HCT-116 and HT-29 colon cancer cells, thereby reducing A4CT2 methylation levels, promoting gene transcription, significantly down-regulating the expression of key genes in the Wnt signaling pathway downstream of A4CT2, and down-regulating the expression of Wnt/β-catenin signaling pathway proteins in HCT-116 cells, thereby blocking cells in the G1 phase and inducing apoptosis.	[121]
2	38748-32-2	Triptolide	diterpene lactone	Triterpenoids and epoxyditerpene lactones from Radix Rehmanniae, family Weigelaeaceae	Inhibits β-catenin entry into the nucleus in the Wnt/β-catenin signaling pathway, reduces downstream target gene LEF/TCF expression, inhibits cell transcription and translation, causes G1 phase block in colon cancer HT29 and SW480 cells, and inhibits cell proliferation in a dose- and time-dependent manner	[122]
3	518-34-3	6,6′,7,12-Tetramethoxy-2,2′-di	Bisbenzy-isoquinoline alkaloids	A dibenzylisoquinoline alkaloid of the plant Fangqi, family Fangqiidae.	Inhibition of LEF/TCF4 and c-Myc/Max transcriptional activity and β-catenin protein expression in the Wnt/β-catenin signaling pathway, thereby inhibiting the proliferation of colon cancer HCT116 cells	[123]
4	14197-60-5	Ginsenoside Rg3	tetracyclic triterpenoid saponin	A tetracyclic triterpenoid saponin from Ginseng, family Ginseng	It inhibits β-catenin/TCF4 transcriptional activity and downstream c-Myc protein expression by blocking the nuclear translocation of β-catenin, thereby inhibiting the proliferation of colon cancer HCT116 and SW480 cells, inducing apoptosis, reducing β-catenin phosphorylation and decreasing β-catenin and c-Myc protein expression.	[124]
5	55297-87-5	Falcarindiol	polyacetylene oxide	A high content of kynurenine compounds in Northeastern prickly ginseng of the genus Pentaphyllaceae.	Inhibition of cell proliferation of colon cancer HCT116 cells in the G2/M phase by suppressing the expression of β-catenin, cyclinD1 and c-Myc in the Wnt/β-catenin pathway	[125]
6	57-24-9	Strychnine	alkaloid	An alkaloid isolated from strychnine	Inhibition of cell proliferation of colon cancer HCT116 cells in the G2/M phase by suppressing the expression of β-catenin, cyclinD1 and c-Myc in the Wnt/β-catenin pathway	[126]
7	518-17-2	Evodiamine	alkaloid	An alkaloid from Cornus officinalis, family Rutaceae	Reduced expression of Wnt and β-catenin mRNA and consequent inhibition of Wnt/β-catenin downstream of vascular endothelial growth factor	[127]
8	528-43-8	Magnolol	Dimeric phenylpropanoids	A new lignan-like compound from the Magnoliaceae thicket	Enhances the expression of caspase 3 protein and promotes apoptosis	[128]
9	632-85-9	Wogonin	flavonoid	A flavonoid from plants such as Scutellaria baicalensis	Modulation of the expression of Wnt/β-catenin signal pathway-related proteins β-catenin, survivin, GSK-3β and Bcl-2-related X protein blocks cells in the G1 phase.	[129]
10	20958-18-3	Dihydrotanshinone	Fat-soluble phenanthrenequinone compounds	Salvia Miltiorrhiza Root Extract	Inhibition of Wnt/β-catenin signaling by down-regulation of β-catenin and/or c-Myc expression to suppress proliferation of colon cancer cells.	[130]
11	2086-83-1	Berberine	Quaternary isoquinoline alkaloids	A quaternary ammonium alkaloid from the Chinese medicine Huanglian	Inhibition of Wnt/β-catenin signaling by down-regulation of β-catenin and/or c-Myc expression to suppress proliferation of colon cancer cells.	[58]
12	76296-75-8	Polyphyllin VII	steroidal saponin	Steroidal saponins extracted from the lily family Chrysanthemum.	Induces apoptosis by upregulating the expression of activated caspase-3, caspase-8, caspase-9 and Bcl-2-related X proteins and downregulating Bcl-2 expression	[131]
13	2752-65-0	GAMBOGIC ACID	flavonoid	The main active ingredient of Garcinia Cambogia, family Garciniaceae.	Induced apoptosis by up-regulation of activated caspase-3, activated caspase-9, Beclin 1, microtubule-associated protein 1 light chain 3-II/microtubule-associated protein 1 light chain 3-I levels and down-regulation of β-catenin, c-Myc levels	[132]
14	126-19-2	Sarsasapogenin	saponin	Saponin elements in the Chinese medicine Zhi Mu	Decrease the relative expression of β-catenin, c-Myc and TCF4 proteins and increase the relative expression of epithelial calmodulin protein, inhibit cell proliferation and induce apoptosis	[133]
15	491-70-3	Luteolin	flavonoid	A flavonoid found in plants such as honeysuckle and fruits and vegetables such as peppers	Enhanced expression of epithelial calcineurin, inhibition of Twist, β-catenin, TCF4 and MMP-2 expression, and inhibition of cell invasion and metastasis.	[134]
16	520-36-5	Apigenin	flavonoid	A flavonoid found in fruits and vegetables such as celery.	Reduces β-catenin, mRNA and protein levels, promotes β-catenin phosphorylation, inhibits β-catenin nuclear translocation, which in turn reduces the expression of Wnt/β-catenin downstream metastasis-related protein cyclooxygenase 2, human filament protein formation enhancer 1, and MMP-2, thereby inhibiting colorectal cancer value-added.	[135]
17	518-82-1	Emodin;	flavonoid	A mono-anthracene-nuclear dihydroxyanthraquinone derivative from rhubarb and other plants	Inhibition of graft tumour growth and microangiogenesis through downregulation of pro-angiogenic factor expression in the Wnt/β-catenin signal pathway.	[136]
18	28957-04-2	Oridonin	Kaurane-type diterpenoids	Extracts of the plant Camellia sinensis, family Labiatae	Inhibition of Wnt/β-catenin signaling by down-regulation of β-catenin and/or c-Myc expression to suppress proliferation of colon cancer cells.	[137]
19	501-36-0	Resveratrol	Non-flavonoid polypheolic organic compounds	Polyphenols from plants such as grapes	It can inhibit the transcriptional activity of TCF4/LEF reporter plasmid and reduce the expression of β-catenin protein and mRNA. Reduced phosphorylated GSK-3β protein levels, β-catenin mRNA expression levels and β-catenin/TCF4 transcriptional activity inhibited proliferation of colon cancer HCT116 cells, blocked cells in G1 phase and promoted apoptosis.	[138]
20	88495-63-0	Artesunate	diterpene lactone	A derivative of artemisinin from Artemisia annua, family Asteraceae	Inhibition of Wnt/β-catenin signaling by down-regulation of β-catenin and/or c-Myc expression to suppress proliferation of colon cancer cells.	[139]

## Data Availability

No new data were created or analyzed in this study. Data sharing is not applicable to this article.

## References

[1] Swain B.C., Subadini S., Rout J., Mishra P.P., Sahoo H., Tripathy U., Sakshi (2020). Biophysical study on complex formation between β-Lactoglobulin and vitamin B12. Food Chem..

[2] Miceli M., Bontempo P., Nebbioso A., Altucci L. (2014). Natural compounds in epigenetics: A current view. Food Chem. Toxicol..

[3] Allis C.D., Jenuwein T. (2016). The molecular hallmarks of epigenetic control. Nat. Rev. Genet..

[4] Beumer J.H., Tawbi H. (2010). Role of histone deacetylases and their inhibitors in cancer biology and treatment. Curr. Clin. Pharmacol..

[5] Feinberg A.P., Tycko B. (2004). The history of cancer epigenetics. Nat. Rev. Cancer.

[6] Yu J., Liu D., Sun X., Yang K., Yao J., Cheng C., Wang C., Zheng J. (2019). CDX2 inhibits the proliferation and tumor formation of colon cancer cells by suppressing Wnt/β-catenin signaling via transactivation of GSK-3β and Axin2 expression. Cell Death Dis..

[7] Li X.Y., Zhan X.R., Liu X.M., Wang X.C. (2011). CREB is a regulatory target for the protein kinase Akt/PKB in the differentiation of pancreatic ductal cells into islet β-cells mediated by hepatocyte growth factor. Biochem. Biophys. Res. Commun..

[8] Cao H., Song S., Zhang H., Zhang Y., Qu R., Yang B., Jing Y., Hu T., Yan F., Wang B. (2013). Chemopreventive effects of berberine on intestinal tumor development in Apcmin/+ mice. BMC Gastroenterol..

[9] Sandig B.V., Brand K., Herwig S., Lukas J., Bartek J., Strauss M. (1997). p16 and p53 genes transferred with the help of adenovirus to induce apoptic tumor cell death. Ugeskr. Laeger.

[10] Lee C.W., Ito K., Ito Y. (2010). Role of RUNX3 in bone morphogenetic protein signaling in colorectal cancer. Cancer Res..

[11] Aladhraei M., Al-Salami E., Poungvarin N., Suwannalert P. (2019). The roles of p53 and XPO1 on colorectal cancer progression in Yemeni patients. J. Gastrointest. Oncol..

[12] Cedar H., Bergman Y. (2009). Linking DNA methylation and histone modification: Patterns and paradigms. Nat. Rev. Genet..

[13] Irshad R., Husain M. (2021). Natural products in the reprogramming of cancer epigenetics. Toxicol. Appl. Pharmacol..

[14] Kanherkar R.R., Bhatia-Dey N., Csoka A.B. (2014). Epigenetics across the human lifespan. Front. Cell Dev. Biol..

[15] Watanabe O., Imamura H., Shimizu T., Kinoshita J., Okabe T., Hirano A., Yoshimatsu K., Konno S., Aiba M., Ogawa K. (2004). Expression of twist and wnt in human breast cancer. Anticancer. Res..

[16] Shi Y., Lan F., Matson C., Mulligan P., Whetstine J.R., Cole P.A., Casero R.A., Shi Y. (2004). Histone demethylation mediated by the nuclear amine oxidase homolog LSD1. Cell.

[17] Sakane C., Okitsu T., Wada A., Sagami H., Shidoji Y. (2014). Inhibition of lysine-specific demethylase 1 by the acyclic diterpenoid geranylgeranoic acid and its derivatives. Biochem. Biophys. Res. Commun..

[18] Biswas S., Rao C.M. (2018). Epigenetic tools (The Writers, The Readers and The Erasers) and their implications in cancer therapy. Eur. J. Pharmacol..

[19] Murray-Zmijewski F., Slee E.A., Lu X. (2008). A complex barcode underlies the heterogeneous response of p53 to stress. Nat. Rev. Mol. Cell Biol..

[20] Jiang L., Kon N., Li T., Wang S.J., Su T., Hibshoosh H., Baer R., Gu W. (2015). Ferroptosis as a p53-mediated activity during tumour suppression. Nature.

[21] Vousden K.H., Lane D.P. (2007). p53 in health and disease. Nat. Rev. Mol. Cell Biol..

[22] Liebl M.C., Hofmann T.G. (2021). The Role of p53 Signaling in Colorectal Cancer. Cancers.

[23] Li J.X., Liu H.L. (2004). The relationship of DNA methylation and histone methylation. Hereditas.

[24] Alelu-Paz R., Ashour N., Gonzalez-Corpas A., Ropero S. (2012). DNA methylation, histone modifications, and signal transduction pathways: A close relationship in malignant gliomas pathophysiology. J. Signal Transduct..

[25] Rose N.R., Klose R.J. (2014). Understanding the relationship between DNA methylation and histone lysine methylation. Biochim. Biophys. Acta.

[26] Cheng X., Xu X., Chen D., Zhao F., Wang W. (2019). Therapeutic potential of targeting the Wnt/β-catenin signaling pathway in colorectal cancer. Biomed. Pharmacother..

[27] Lomenick B., Jung G., Wohlschlegel J.A., Huang J. (2011). Target identification using drug affinity responsive target stability (DARTS). Curr. Protoc. Chem. Biol..

[28] Lomenick B., Hao R., Jonai N., Chin R.M., Aghajan M., Warburton S., Wang J., Wu R.P., Gomez F., Loo J.A. (2009). Target identification using drug affinity responsive target stability (DARTS). Proc. Natl. Acad. Sci. USA.

[29] Lamouille S., Xu J., Derynck R. (2014). Molecular mechanisms of epithelial-mesenchymal transition. Nat. Rev. Mol. Cell Biol..

[30] Lee Y.M., Kim S.H., Kim M.S., Kim D.C., Lee E.H., Lee J.S., Lee S.H., Kim Y.Z. (2020). Epigenetic Role of Histone Lysine Methyltransferase and Demethylase on the Expression of Transcription Factors Associated with the Epithelial-to-Mesenchymal Transition of Lung Adenocarcinoma Metastasis to the Brain. Cancers.

[31] Chatterjee B., Ghosh K., Suresh L., Kanade S.R. (2019). Curcumin ameliorates PRMT5-MEP50 arginine methyltransferase expression by decreasing the Sp1 and NF-YA transcription factors in the A549 and MCF-7 cells. Mol. Cell Biochem..

[32] Hampsey M., Reinberg D. (2003). Tails of intrigue: Phosphorylation of RNA polymerase II mediates histone methylation. Cell.

[33] Bradley W.D., Arora S., Busby J., Balasubramanian S., Gehling V.S., Nasveschuk C.G., Vaswani R.G., Yuan C.C., Hatton C., Zhao F. (2014). EZH2 inhibitor efficacy in non-Hodgkin’s lymphoma does not require suppression of H3K27 monomethylation. Chem. Biol..

[34] Kung P.P., Rui E., Bergqvist S., Bingham P., Braganza J., Collins M., Cui M., Diehl W., Dinh D., Fan C. (2016). Correction to Design and Synthesis of Pyridone-Containing 3,4-Dihydroisoquinoline-1(2H)-ones as a Novel Class of Enhancer of Zeste Homolog 2 (EZH2) Inhibitors. J. Med. Chem..

[35] Izquierdo-Torres E., Hernandez-Oliveras A., Meneses-Morales I., Rodriguez G., Fuentes-Garcia G., Zarain-Herzberg A. (2019). Resveratrol up-regulates ATP2A3 gene expression in breast cancer cell lines through epigenetic mechanisms. Int. J. Biochem. Cell Biol..

[36] Rice J.C., Allis C.D. (2001). Histone methylation versus histone acetylation: New insights into epigenetic regulation. Curr. Opin. Cell Biol..

[37] Campbell J.E., Kuntz K.W., Knutson S.K., Warholic N.M., Keilhack H., Wigle T.J., Raimondi A., Klaus C.R., Rioux N., Yokoi A. (2015). EPZ011989, A Potent, Orally-Available EZH2 Inhibitor with Robust in Vivo Activity. ACS Med. Chem. Lett..

[38] Zhang L., Liu Z., Ma W., Wang B. (2013). The landscape of histone acetylation involved in epithelial-mesenchymal transition in lung cancer. J. Cancer Res. Ther..

[39] He W., Yu Y., Huang W., Feng G., Li J. (2020). The Pseudogene DUXAP8 Promotes Colorectal Cancer Cell Proliferation, Invasion, and Migration by Inducing Epithelial-Mesenchymal Transition Through Interacting with EZH2 and H3K27me3. Onco Targets Ther..

[40] Wissmann M., Yin N., Muller J.M., Greschik H., Fodor B.D., Jenuwein T., Vogler C., Schneider R., Gunther T., Buettner R. (2007). Cooperative demethylation by JMJD2C and LSD1 promotes androgen receptor-dependent gene expression. Nat. Cell Biol..

[41] Ruthenburg A.J., Allis C.D., Wysocka J. (2007). Methylation of lysine 4 on histone H3: Intricacy of writing and reading a single epigenetic mark. Mol. Cell.

[42] Hossain S., Liu Z., Wood R.J. (2021). Association between histone deacetylase activity and vitamin D-dependent gene expressions in relation to sulforaphane in human colorectal cancer cells. J. Sci. Food Agric..

[43] Chung J.W., Noh E.J., Zhao H.L., Sim J.S., Ha Y.W., Shin E.M., Lee E.B., Cheong C.S., Kim Y.S. (2008). Anti-inflammatory activity of prosapogenin methyl ester of platycodin D via nuclear factor-kappaB pathway inhibition. Biol. Pharm. Bull..

[44] Jiang B.H., Liu L.Z. (2009). PI3K/PTEN signaling in angiogenesis and tumorigenesis. Adv. Cancer Res..

[45] Huang P., Sun L.Y., Zhang Y.Q. (2019). A Hopeful Natural Product, Pristimerin, Induces Apoptosis, Cell Cycle Arrest, and Autophagy in Esophageal Cancer Cells. Anal Cell Pathol.

[46] Song L., Li Z.Y., Liu W.P., Zhao M.R. (2015). Crosstalk between Wnt/β-catenin and Hedgehog/Gli signaling pathways in colon cancer and implications for therapy. Cancer Biol. Ther..

[47] Gao F., Zhang Y., Wang S., Liu Y., Zheng L., Yang J., Huang W., Ye Y., Luo W., Xiao D. (2014). Hes1 is involved in the self-renewal and tumourigenicity of stem-like cancer cells in colon cancer. Sci. Rep..

[48] Liu L.C., Tsao T.C., Hsu S.R., Wang H.C., Tsai T.C., Kao J.Y., Way T.D. (2012). EGCG inhibits transforming growth factor-β-mediated epithelial-to-mesenchymal transition via the inhibition of Smad2 and Erk1/2 signaling pathways in nonsmall cell lung cancer cells. J. Agric. Food Chem..

[49] Liu Z., Duan Z.J., Chang J.Y., Zhang Z.F., Chu R., Li Y.L., Dai K.H., Mo G.Q., Chang Q.Y. (2019). Retraction: Sinomenine Sensitizes Multidrug-Resistant Colon Cancer Cells (Caco-2) to Doxorubicin by Downregulation of MDR-1 Expression. PLoS ONE.

[50] Wang K., Liu W., Xu Q., Gu C., Hu D. (2021). Tenacissoside G synergistically potentiates inhibitory effects of 5-fluorouracil to human colorectal cancer. Phytomedicine.

[51] Bai Y., Wang X., Cai M., Ma C., Xiang Y., Hu W., Zhou B., Zhao C., Dai X., Li X. (2021). Cinobufagin suppresses colorectal cancer growth via STAT3 pathway inhibition. Am. J. Cancer Res..

[52] Wang K., Huang W., Sang X., Wu X., Shan Q., Tang D., Xu X., Cao G. (2020). Atractylenolide I inhibits colorectal cancer cell proliferation by affecting metabolism and stemness via AKT/mTOR signaling. Phytomedicine.

[53] Kimura Y., Sumiyoshi M., Taniguchi M. (2023). Geniposide prevents tumor growth by inhibiting colonic interleukin-1β and monocyte chemoattractant protein-1 via down-regulated expression of cyclooxygenase-2 and thymocyte selection-associated high mobility box proteins TOX/TOX2 in azoxymethane/dextran sulfate sodium-treated mice. Int. Immunopharmacol..

[54] Liu B., Jin J., Zhang Z., Zuo L., Jiang M., Xie C. (2019). Shikonin exerts antitumor activity by causing mitochondrial dysfunction in hepatocellular carcinoma through PKM2-AMPK-PGC1alpha signaling pathway. Biochem. Cell Biol..

[55] Cai H., Hu H. (2021). Costunolide Induces Apoptosis of K562/ADR Cells through PI3K/AKT Pathway. Zhongguo Shi Yan Xue Ye Xue Za Zhi.

[56] He F., Xiong W., Wang Y., Li L., Liu C., Yamagami T., Taketo M.M., Zhou C., Chen Y. (2011). Epithelial Wnt/β-catenin signaling regulates palatal shelf fusion through regulation of Tgfβ3 expression. Dev. Biol..

[57] Li X.Y., Feng Y.Y., Dan W., Pan D., Zhang G.F., Wang X.L., Hou G.J. (2016). Study on the influence of curcumin on chemosensitivity of nephroblastoma cells. Asian Pac. J. Trop. Med..

[58] Wu K., Yang Q., Mu Y., Zhou L., Liu Y., Zhou Q., He B. (2012). Berberine inhibits the proliferation of colon cancer cells by inactivating Wnt/β-catenin signaling. Int. J. Oncol..

[59] Tan M., Luo H., Lee S., Jin F., Yang J.S., Montellier E., Buchou T., Cheng Z., Rousseaux S., Rajagopal N. (2011). Identification of 67 histone marks and histone lysine crotonylation as a new type of histone modification. Cell.

[60] Li D., Yang Y., Wang S., He X., Liu M., Bai B., Tian C., Sun R., Yu T., Chu X. (2021). Role of acetylation in doxorubicin-induced cardiotoxicity. Redox Biol..

[61] Marks P., Rifkind R.A., Richon V.M., Breslow R., Miller T., Kelly W.K. (2001). Histone deacetylases and cancer: Causes and therapies. Nat. Rev. Cancer.

[62] Li X., Peterson Y.K., Inks E.S., Himes R.A., Li J., Zhang Y., Kong X., Chou C.J. (2018). Class I HDAC Inhibitors Display Different Antitumor Mechanism in Leukemia and Prostatic Cancer Cells Depending on Their p53 Status. J. Med. Chem..

[63] Singh B.N., Zhang G., Hwa Y.L., Li J., Dowdy S.C., Jiang S.W. (2010). Nonhistone protein acetylation as cancer therapy targets. Expert. Rev. Anticancer. Ther..

[64] Mottamal M., Zheng S., Huang T.L., Wang G. (2015). Histone deacetylase inhibitors in clinical studies as templates for new anticancer agents. Molecules.

[65] Zhao S., Cheng L., Gao Y., Zhang B., Zheng X., Wang L., Li P., Sun Q., Li H. (2019). Plant HP1 protein ADCP1 links multivalent H3K9 methylation readout to heterochromatin formation. Cell Res..

[66] Liu D., Zhang H., Cong J., Cui M., Ma M., Zhang F., Sun H., Chen C. (2020). H3K27 acetylation-induced lncRNA EIF3J-AS1 improved proliferation and impeded apoptosis of colorectal cancer through miR-3163/YAP1 axis. J. Cell Biochem..

[67] Wang S., Zang C., Xiao T., Fan J., Mei S., Qin Q., Wu Q., Li X., Xu K., He H.H. (2016). Modeling cis-regulation with a compendium of genome-wide histone H3K27ac profiles. Genome Res..

[68] Li Y., Seto E. (2016). HDACs and HDAC Inhibitors in Cancer Development and Therapy. Cold Spring Harb. Perspect. Med..

[69] Salvador L.A., Luesch H. (2012). Discovery and mechanism of natural products as modulators of histone acetylation. Curr. Drug Targets.

[70] Chen K., Zhang F., Ding J., Liang Y., Zhan Z., Zhan Y., Chen L.H., Ding Y. (2017). Histone Methyltransferase SETDB1 Promotes the Progression of Colorectal Cancer by Inhibiting the Expression of TP53. J. Cancer.

[71] Mitani Y., Oue N., Hamai Y., Aung P.P., Matsumura S., Nakayama H., Kamata N., Yasui W. (2005). Histone H3 acetylation is associated with reduced p21(WAF1/CIP1) expression by gastric carcinoma. J. Pathol..

[72] Couture J.F., Trievel R.C. (2006). Histone-modifying enzymes: Encrypting an enigmatic epigenetic code. Curr. Opin. Struct. Biol..

[73] Zhang K., Dent S.Y. (2005). Histone modifying enzymes and cancer: Going beyond histones. J. Cell Biochem..

[74] Audia J.E., Campbell R.M. (2016). Histone Modifications and Cancer. Cold Spring Harb. Perspect. Biol..

[75] Kuo M.H., Allis C.D. (1998). Roles of histone acetyltransferases and deacetylases in gene regulation. Bioessays.

[76] Danielsen S.A., Eide P.W., Nesbakken A., Guren T., Leithe E., Lothe R.A. (2015). Portrait of the PI3K/AKT pathway in colorectal cancer. Biochim. Biophys. Acta.

[77] Zhang H., Li L., Yuan C., Wang C., Gao T., Zheng Z. (2020). MiR-489 inhibited the development of gastric cancer via regulating HDAC7 and PI3K/AKT pathway. World J. Surg. Oncol..

[78] Yarushkin A.A., Mazin M.E., Yunusova A.Y., Korchagina K.V., Pustylnyak Y.A., Prokopyeva E.A., Pustylnyak V.O. (2018). CAR-mediated repression of Cdkn1a(p21) is accompanied by the Akt activation. Biochem. Biophys. Res. Commun..

[79] Nural-Guvener H., Zakharova L., Feehery L., Sljukic S., Gaballa M. (2015). Anti-Fibrotic Effects of Class I HDAC Inhibitor, Mocetinostat Is Associated with IL-6/Stat3 Signaling in Ischemic Heart Failure. Int. J. Mol. Sci..

[80] Akone S.H., Ntie-Kang F., Stuhldreier F., Ewonkem M.B., Noah A.M., Mouelle S.E.M., Muller R. (2020). Natural Products Impacting DNA Methyltransferases and Histone Deacetylases. Front. Pharmacol..

[81] Yoshida M., Kijima M., Akita M., Beppu T. (1990). Potent and specific inhibition of mammalian histone deacetylase both in vivo and in vitro by trichostatin A. J. Biol. Chem..

[82] Plumb J.A., Finn P.W., Williams R.J., Bandara M.J., Romero M.R., Watkins C.J., La Thangue N.B., Brown R. (2003). Pharmacodynamic response and inhibition of growth of human tumor xenografts by the novel histone deacetylase inhibitor PXD101. Mol. Cancer Ther..

[83] Scuto A., Kirschbaum M., Kowolik C., Kretzner L., Juhasz A., Atadja P., Pullarkat V., Bhatia R., Forman S., Yen Y. (2008). The novel histone deacetylase inhibitor, LBH589, induces expression of DNA damage response genes and apoptosis in Ph- acute lymphoblastic leukemia cells. Blood.

[84] Arts J., King P., Marien A., Floren W., Belien A., Janssen L., Pilatte I., Roux B., Decrane L., Gilissen R. (2009). JNJ-26481585, a novel "second-generation" oral histone deacetylase inhibitor, shows broad-spectrum preclinical antitumoral activity. Clin. Cancer Res..

[85] Phiel C.J., Zhang F., Huang E.Y., Guenther M.G., Lazar M.A., Klein P.S. (2001). Histone deacetylase is a direct target of valproic acid, a potent anticonvulsant, mood stabilizer, and teratogen. J. Biol. Chem..

[86] Aviram A., Zimrah Y., Shaklai M., Nudelman A., Rephaeli A. (1994). Comparison between the effect of butyric acid and its prodrug pivaloyloxymethylbutyrate on histones hyperacetylation in an HL-60 leukemic cell line. Int. J. Cancer.

[87] DiGiuseppe J.A., Weng L.J., Yu K.H., Fu S., Kastan M.B., Samid D., Gore S.D. (1999). Phenylbutyrate-induced G1 arrest and apoptosis in myeloid leukemia cells: Structure-function analysis. Leukemia.

[88] Giannini G., Vesci L., Battistuzzi G., Vignola D., Milazzo F.M., Guglielmi M.B., Barbarino M., Santaniello M., Fanto N., Mor M. (2014). ST7612AA1, a thioacetate-ω(γ-lactam carboxamide) derivative selected from a novel generation of oral HDAC inhibitors. J. Med. Chem..

[89] Hu E., Dul E., Sung C.M., Chen Z., Kirkpatrick R., Zhang G.F., Johanson K., Liu R., Lago A., Hofmann G. (2003). Identification of novel isoform-selective inhibitors within class I histone deacetylases. J. Pharmacol. Exp. Ther..

[90] Goncalves P., Martel F. (2013). Butyrate and colorectal cancer: The role of butyrate transport. Curr. Drug Metab..

[91] De Souza C., Chatterji B.P. (2015). HDAC Inhibitors as Novel Anti-Cancer Therapeutics. Recent. Pat. Anticancer. Drug Discov..

[92] Meng F., Sun G., Zhong M., Yu Y., Brewer M.A. (2013). Anticancer efficacy of cisplatin and trichostatin A or 5-aza-2’-deoxycytidine on ovarian cancer. Br. J. Cancer.

[93] Saijo K., Imamura J., Narita K., Oda A., Shimodaira H., Katoh T., Ishioka C. (2015). Biochemical, biological and structural properties of romidepsin (FK228) and its analogs as novel HDAC/PI3K dual inhibitors. Cancer Sci..

[94] Marcu M.G., Jung Y.J., Lee S., Chung E.J., Lee M.J., Trepel J., Neckers L. (2006). Curcumin is an inhibitor of p300 histone acetylatransferase. Med. Chem..

[95] Collins H.M., Abdelghany M.K., Messmer M., Yue B., Deeves S.E., Kindle K.B., Mantelingu K., Aslam A., Winkler G.S., Kundu T.K. (2013). Differential effects of garcinol and curcumin on histone and p53 modifications in tumour cells. BMC Cancer.

[96] Kuttan R., Sudheeran P.C., Josph C.D. (1987). Turmeric and curcumin as topical agents in cancer therapy. Tumori.

[97] Santer F.R., Hoschele P.P., Oh S.J., Erb H.H., Bouchal J., Cavarretta I.T., Parson W., Meyers D.J., Cole P.A., Culig Z. (2011). Inhibition of the acetyltransferases p300 and CBP reveals a targetable function for p300 in the survival and invasion pathways of prostate cancer cell lines. Mol. Cancer Ther..

[98] Santoni M., Massari F., Del Re M., Ciccarese C., Piva F., Principato G., Montironi R., Santini D., Danesi R., Tortora G. (2015). Investigational therapies targeting signal transducer and activator of transcription 3 for the treatment of cancer. Expert. Opin. Investig. Drugs.

[99] Lao V.V., Grady W.M. (2011). Epigenetics and colorectal cancer. Nat. Rev. Gastroenterol. Hepatol..

[100] Zeng S., Chen L., Sun Q., Zhao H., Yang H., Ren S., Liu M., Meng X., Xu H. (2021). Scutellarin ameliorates colitis-associated colorectal cancer by suppressing Wnt/β-catenin signaling cascade. Eur. J. Pharmacol..

[101] Yang L., Zhu T., Ye H., Shen Y., Li Z., Chen L., Wang C., Chen X., Zhao H., Xiang Y. (2021). Gracillin shows potent efficacy against colorectal cancer through inhibiting the STAT3 pathway. J. Cell Mol. Med..

[102] Baker S.P., Phillips J., Anderson S., Qiu Q., Shabanowitz J., Smith M.M., Yates J.R., Hunt D.F., Grant P.A. (2010). Histone H3 Thr 45 phosphorylation is a replication-associated post-translational modification in S. cerevisiae. Nat. Cell Biol..

[103] Thorsness P.E., Koshland D.E. (1987). Inactivation of isocitrate dehydrogenase by phosphorylation is mediated by the negative charge of the phosphate. J. Biol. Chem..

[104] North J.A., Simon M., Ferdinand M.B., Shoffner M.A., Picking J.W., Howard C.J., Mooney A.M., van Noort J., Poirier M.G., Ottesen J.J. (2014). Histone H3 phosphorylation near the nucleosome dyad alters chromatin structure. Nucleic Acids Res..

[105] Huang D., Cui L., Ahmed S., Zainab F., Wu Q., Wang X., Yuan Z. (2019). An overview of epigenetic agents and natural nutrition products targeting DNA methyltransferase, histone deacetylases and microRNAs. Food Chem. Toxicol..

[106] Verdone L., Agricola E., Caserta M., Di Mauro E. (2006). Histone acetylation in gene regulation. Brief. Funct. Genom. Proteomic.

[107] Hu X.Q., Su S.B. (2017). An overview of epigenetics in Chinese medicine researches. Chin. J. Integr. Med..

[108] Liu D., Yan H., Kong Y., You Y., Li Y., Wang L., Tong Y., Wang J. (2018). Preparation of Colon-Targeted Acetylharpagide Tablets and its Release Properties in vivo and in vitro. Front. Pharmacol..

[109] Yurasakpong L., Nantasenamat C., Nobsathian S., Chaithirayanon K., Apisawetakan S. (2021). Betulinic Acid Modulates the Expression of HSPA and Activates Apoptosis in Two Cell Lines of Human Colorectal Cancer. Molecules.

[110] Yan W., Bai Z., Wang J., Li X., Chi B., Chen X. (2017). ANP32A modulates cell growth by regulating p38 and Akt activity in colorectal cancer. Oncol. Rep..

[111] Hu S., Liu Y., Guan S., Qiu Z., Liu D. (2022). Natural products exert anti-tumor effects by regulating exosomal ncRNA. Front. Oncol..

[112] Wang H., Geng Q.R., Wang L., Lu Y. (2012). Curcumin potentiates antitumor activity of L-asparaginase via inhibition of the AKT signaling pathway in acute lymphoblastic leukemia. Leuk. Lymphoma.

[113] Hanikoglu A., Hanikoglu F., Ozben T. (2018). Natural Product Inhibitors of Histone Deacetylases as New Anticancer Agents. Curr. Protein Pept. Sci..

[114] Jin Y., Liu T., Luo H., Liu Y., Liu D. (2022). Targeting Epigenetic Regulatory Enzymes for Cancer Therapeutics: Novel Small-Molecule Epidrug Development. Front. Oncol..

[115] Cuttini E., Goi C., Pellarin E., Vida R., Brancolini C. (2023). HDAC4 in cancer: A multitasking platform to drive not only epigenetic modifications. Front. Mol. Biosci..

[116] Baylin S.B., Jones P.A. (2011). A decade of exploring the cancer epigenome—Biological and translational implications. Nat. Rev. Cancer.

[117] Baba Y., Murata A., Watanabe M., Baba H. (2014). Clinical implications of the LINE-1 methylation levels in patients with gastrointestinal cancer. Surg. Today.

[118] Tan S.H., Barker N. (2018). Wnt Signaling in Adult Epithelial Stem Cells and Cancer. Prog. Mol. Biol. Transl. Sci..

[119] Zhao H., Ming T., Tang S., Ren S., Yang H., Liu M., Tao Q., Xu H. (2022). Wnt signaling in colorectal cancer: Pathogenic role and therapeutic target. Mol. Cancer.

[120] Ghate N.B., Kim S., Spiller E., Kim S., Shin Y., Rhie S.K., Smbatyan G., Lenz H.J., Mumenthaler S.M., An W. (2021). VprBP directs epigenetic gene silencing through histone H2A phosphorylation in colon cancer. Mol. Oncol..

[121] Pabla B., Bissonnette M., Konda V.J. (2015). Colon cancer and the epidermal growth factor receptor: Current treatment paradigms, the importance of diet, and the role of chemoprevention. World J. Clin. Oncol..

[122] Zhang C., Cui G.H., Liu F., Wu Q.L., Chen Y. (2006). Inhibitory effect of triptolide on lymph node metastasis in patients with non-Hodgkin lymphoma by regulating SDF-1/CXCR4 axis in vitro. Acta Pharmacol. Sin..

[123] Meng L.H., Zhang H., Hayward L., Takemura H., Shao R.G., Pommier Y. (2004). Tetrandrine induces early G1 arrest in human colon carcinoma cells by down-regulating the activity and inducing the degradation of G1-S-specific cyclin-dependent kinases and by inducing p53 and p21Cip1. Cancer Res..

[124] He B.C., Gao J.L., Luo X., Luo J., Shen J., Wang L., Zhou Q., Wang Y.T., Luu H.H., Haydon R.C. (2011). Ginsenoside Rg3 inhibits colorectal tumor growth through the down-regulation of Wnt/ss-catenin signaling. Int. J. Oncol..

[125] Wu K., Wang C.Z., Yuan C.S., Huang W.H. (2018). Oplopanax horridus: Phytochemistry and Pharmacological Diversity and Structure-Activity Relationship on Anticancer Effects. Evid. Based Complement. Altern. Med..

[126] Luo W., Wang X., Zheng L., Zhan Y., Zhang D., Zhang J., Zhang Y. (2013). Brucine suppresses colon cancer cells growth via mediating KDR signalling pathway. J. Cell Mol. Med..

[127] Chien C.C., Wu M.S., Shen S.C., Ko C.H., Chen C.H., Yang L.L., Chen Y.C. (2014). Activation of JNK contributes to evodiamine-induced apoptosis and G2/M arrest in human colorectal carcinoma cells: A structure-activity study of evodiamine. PLoS ONE.

[128] Jiang J., Griffin J.D. (2010). Wnt/β-catenin Pathway Modulates the Sensitivity of the Mutant FLT3 Receptor Kinase Inhibitors in a GSK-3β Dependent Manner. Genes. Cancer.

[129] Kim S.J., Kim H.J., Kim H.R., Lee S.H., Cho S.D., Choi C.S., Nam J.S., Jung J.Y. (2012). Antitumor actions of baicalein and wogonin in HT-29 human colorectal cancer cells. Mol. Med. Rep..

[130] Taye N., Alam A., Ghorai S., Chatterji D.G., Parulekar A., Mogare D., Singh S., Sengupta P., Chatterjee S., Bhat M.K. (2018). SMAR1 inhibits Wnt/β-catenin signaling and prevents colorectal cancer progression. Oncotarget.

[131] Luo Q., Jia L., Huang C., Qi Q., Jahangir A., Xia Y., Liu W., Shi R., Tang L., Chen Z. (2022). Polyphyllin I Promotes Autophagic Cell Death and Apoptosis of Colon Cancer Cells via the ROS-Inhibited AKT/mTOR Pathway. Int. J. Mol. Sci..

[132] Song C., Pan B., Yang X., Tang W. (2021). Polyphyllin VII suppresses cell proliferation, the cell cycle and cell migration in colorectal cancer. Oncol. Lett..

[133] Mandlik D.S., Mandlik S.K., Patel S. (2021). Protective effect of sarsasapogenin in TNBS induced ulcerative colitis in rats associated with downregulation of pro-inflammatory mediators and oxidative stress. Immunopharmacol. Immunotoxicol..

[134] Yoo H.S., Won S.B., Kwon Y.H. (2022). Luteolin Induces Apoptosis and Autophagy in HCT116 Colon Cancer Cells via p53-Dependent Pathway. Nutr. Cancer.

[135] Ji Q., Liu X., Fu X., Zhang L., Sui H., Zhou L., Sun J., Cai J., Qin J., Ren J. (2013). Resveratrol inhibits invasion and metastasis of colorectal cancer cells via MALAT1 mediated Wnt/β-catenin signal pathway. PLoS ONE.

[136] Chung J.G., Li Y.C., Lee Y.M., Lin J.P., Cheng K.C., Chang W.C. (2003). Aloe-emodin inhibited N-acetylation and DNA adduct of 2-aminofluorene and arylamine N-acetyltransferase gene expression in mouse leukemia L 1210 cells. Leuk Res.

[137] Gao F.H., Hu X.H., Li W., Liu H., Zhang Y.J., Guo Z.Y., Xu M.H., Wang S.T., Jiang B., Liu F. (2010). Oridonin induces apoptosis and senescence in colorectal cancer cells by increasing histone hyperacetylation and regulation of p16, p21, p27 and c-myc. BMC Cancer.

[138] Liu Y.Z., Wu K., Huang J., Liu Y., Wang X., Meng Z.J., Yuan S.X., Wang D.X., Luo J.Y., Zuo G.W. (2014). The PTEN/PI3K/Akt and Wnt/β-catenin signaling pathways are involved in the inhibitory effect of resveratrol on human colon cancer cell proliferation. Int. J. Oncol..

[139] Riganti C., Doublier S., Viarisio D., Miraglia E., Pescarmona G., Ghigo D., Bosia A. (2009). Artemisinin induces doxorubicin resistance in human colon cancer cells via calcium-dependent activation of HIF-1alpha and P-glycoprotein overexpression. Br. J. Pharmacol..

